# Utility of Circadian Variability Patterns in Differentiating Origins of Premature Ventricular Complexes

**DOI:** 10.1155/2020/7417912

**Published:** 2020-10-30

**Authors:** Mu Chen, Qunshan Wang, Jian Sun, Peng-Pai Zhang, Wei Li, Rui Zhang, Bin-Feng Mo, Tai-Zhong Chen, Juyi Wan, Dong-Zhu Xu, Kazutaka Aonuma, Yi-Gang Li

**Affiliations:** ^1^Department of Cardiology, Xinhua Hospital, School of Medicine, Shanghai Jiao Tong University, Shanghai, China; ^2^Department of Cardiothoracic Surgery, Affiliated Hospital of Southwest Medical University, Luzhou, China; ^3^Cardiovascular Division, Institute of Clinical Medicine, Faculty of Medicine, University of Tsukuba, Tsukuba, Japan

## Abstract

**Background:**

Premature ventricular complexes (PVCs) exhibit circadian fluctuation. We determine if PVCs of different origin exhibit specific circadian patterns.

**Methods:**

We analyzed Holter recordings from patients with monomorphic PVCs who underwent catheter ablation. PVC circadian patterns were classified as fast-heart rate- (HR-) dependent (F-PVC), slow-HR-dependent (S-PVC), or HR-independent (I-PVC). PVC origins were determined intraprocedurally.

**Results:**

In a retrospective cohort of 407 patients, F-PVC and S-PVC typically exhibited diurnal and nocturnal predominance, respectively. Despite decreased circadian fluctuation, I-PVC generally had heavier nocturnal than diurnal burden. PVCs of left anterior fascicle origin were predominantly S-PVC, while those of posterior hemibranch origin were mostly F-PVC. PVCs originating from the aortic sinus of Valsalva (ASV) were predominantly I-PVC, while most PVCs arising from the left ventricular outflow tract (LVOT) were F-PVC. Using a diurnal/nocturnal PVC burden ratio of 0.92 as the cutoff value to distinguish LVOT from ASV origin achieved 97% sensitivity and, as further verification, an accuracy of 89% (16/18) in a prospective cohort of patients with PVCs originating from either ASV or LVOT. In contrast, PVCs originating from right ventricles, such as right ventricular outflow tract, did not show distinct circadian patterns.

**Conclusions:**

The circadian patterns exhibit origin specificity for PVCs arising from left ventricles. An analysis of Holter monitoring provides useful information on PVC localization in ablation procedure planning.

## 1. Introduction

Ventricular arrhythmogenesis exhibits prominent circadian differences. While sudden cardiac death shows diurnal predominance, the lethal events of Brugada syndrome and catecholaminergic polymorphic ventricular tachycardia (CPVT) are highly prevalent at nighttime and afternoon/evening, respectively [1–4]. Circadian vulnerability to ventricular arrhythmias has been linked to various triggering mechanisms, including autonomic nervous activities controlled by the central circadian clock located within the suprachiasmatic nucleus (SCN) of the anterior hypothalamus and the cardiac endogenous rhythmic transcription and translation of ion channels [3–8].

Idiopathic premature ventricular complexes (PVCs), the most common form of ventricular arrhythmias, exhibit robust circadian rhythmicity. Three patterns are recognized according to the correlation of PVC frequency with its corresponding heart rate (HR), i.e., fast-HR-dependent (F-PVC), slow-HR-dependent (S-PVC), and HR-independent (I-PVC) [9, 10], which suggests heterogeneity in arrhythmogenic mechanisms among idiopathic PVCs. Due to the complicated topography of the ventricles, PVCs may be affected by origin-specific pathophysiological conditions, including interventricular and transmural gradients of action potential repolarization, autonomic innervation, and electrotonic interactions among cardiomyocytes and between cardiomyocytes and other cell types. Although 12-lead electrocardiogram (ECG) algorithms are well-established for PVC origin localization [11, 12], it is possible that PVC circadian variability might add useful information when planning ablation procedures. The present study revealed differing circadian variabilities among PVCs of adjacent anatomic origins in left ventricles. The PVC circadian patterns provide further insight on heterogeneous arrhythmogenesis within left fascicular systems.

## 2. Methods

### 2.1. Patient Selection

The study protocol conformed to the ethical guidelines of the 1975 Declaration of Helsinki was approved by the Institutional Review Board committee of Xinhua Hospital, Shanghai, China, and all patients provided informed consent.

We retrospectively reviewed records of patients who underwent radiofrequency ablation of idiopathic PVCs with Holter-documented PVCs >3000 beats/24 h prior to the procedure. Cases with pleomorphic PVCs, sustained ventricular tachycardia (VT), cardiomyopathies, or inconsistent circadian patterns among repeated Holter recordings were excluded.

Because the retrospective evaluation revealed different circadian patterns between PVCs arising from the aortic sinus of Valsalva (ASV, above the aortic valve) and the left ventricular outflow tract (LVOT, below the aortic valve), a second cohort of patients whose PVC origin was predicted to be left-sided with outflow tract morphology by 12-lead ECG [12] was prospectively enrolled to assess the utility of our proposed Holter algorithm in differentiating these two adjacent sources.

### 2.2. PVC Circadian Patterns

The PVC circadian variability pattern was categorized into three types based on Pearson's correlation between hourly PVC counts and hourly average mean HR, as described previously [9], namely, F-PVC if the correlation was significantly positive (*p* < 0.05); S-PVC if the correlation was significantly negative (*p* < 0.05); and I-PVC if no statistical significance was achieved (*p* > 0.05).

For each patient, the hourly PVC counts were ranked and plotted in a line of heatmap (24 gradients) with a 24-hour timeline *x*-axis. The hour with the highest PVC frequency was plotted as red and the hour with lowest PVC frequency as blue. The hours with PVC frequencies in between were presented by colors gradually shifting from red to blue with 24 gradients according to their ranks in 24 hours. In addition, PVC burden was calculated by dividing PVC counts by total heart beats within a certain period. Specifically, diurnal (daytime) PVC burden was calculated from 8 am to 8 pm (12 hours), while nocturnal (nighttime) burden was acquired between 8 pm to 8 am of the next day (12 hours). A diurnal/nocturnal PVC burden ratio was subsequently calculated for PVCs of ASV and LVOT origin.

### 2.3. Mapping and Ablation

No general anesthesia was applied, and none or minimal sedation strategy was used to avoid potential PVC suppression. Intraprocedural mapping and ablation of PVCs were performed. Fifteen sites for PVC origin were as follows: ASV, LVOT, left bundle branch (LBB), left anterior fascicle (LAF), left posterior fascicle (LPF), left anterior papillary muscle (LAPM), left posterior papillary muscle (LPPM), great cardiac vein (GCV), other origins from left ventricles, pulmonary sinus cusp (PSC), the septum of right ventricular outflow tract (RVOT), free wall of RVOT, para-Hisian region, RV inflow tract, and tricuspid annulus (except para-Hisian origin). Undetermined sources with immediate failure of ablation were defined as of “unknown” origin.

### 2.4. Transmembrane Potential of Rabbit Purkinje Cells

Because our human data suggested hemibranch-specific PVC circadian patterns, we conducted a set of rabbit studies to evaluate if LAF and LPF Purkinje cells exhibited different electrophysiological properties, which might contribute to the heterogeneous circadian behaviors of fascicular PVCs. The animal study was approved by the Institutional Animal Care and Use Committee (IACUC) of Xinhua Hospital and was in conformity with the Guide for the Care and Use of Laboratory Animals; all animals received humane care. A total of 15 adult (5-6 months old, 8 males, 3.06–3.32 kg) New Zealand white rabbits were used. As previously described [13], spontaneous transmembrane potentials (TMPs) of Purkinje fibers from both LAF and LPF were recorded, and electrophysiological parameters were compared.

### 2.5. Statistical Analysis

Continuous and ordinal variables are shown as mean ± SEM and nominal variables as *n* (%) as appropriate, unless mentioned specifically. Between-group differences in nominal variables were assessed using Pearson's chi-square tests or Fisher exact tests. Differences in continuous variables among groups were assessed using a one-way ANOVA with Tukey post-tests. Paired Student's *t*-tests were used to compare 2 variables obtained from the same patient or rabbit. A receiver operating characteristic curve was generated for sensitivity and specificity analyses, and Youden's index was applied to determine the optimal cutoff for diurnal/nocturnal PVC burden ratio as a diagnostic test. Statistical analyses were performed using SPSS 23.0 (IBM, Armonk, NY, USA). A 2-sided *p* value < 0.05 was considered statistically significant.

## 3. Results

We retrospectively reviewed data from 509 consecutive patients who underwent radiofrequency catheter ablation of idiopathic PVCs with at least 1 preablation 24-hour Holter recording available between May 2008 and July 2018 at our institution. We excluded patients with severe ischemic or structural heart disease (*n* = 21), pleomorphic PVCs (*n* = 32), and sustained VT (*n* = 41). Among the 111 patients with more than 1 preablation Holter recording, only 8 cases exhibited differing circadian patterns and were subsequently excluded. As shown in Supplemental Figure 1, the majority of cases (*n* = 103, 93%) exhibited consistent patterns among Holter recordings repeated at median 4 months intervals. The high consistency in the PVC circadian pattern among repeat Holter recordings suggests that a single Holter recording might be adequate to reflect the PVC circadian behaviors over a long period of time. Therefore, we further included the remaining 304 patients who had only 1 preablation Holter recording. In total, the retrospective cohort for analyses contained 407 cases, including 196 F-PVC, 67 S-PVC, and 144 I-PVC (Supplemental Figure 2). The patients' demographic data are shown in Table 1. Baseline characteristics were similarly distributed among groups, except patients in the S-PVC group were younger than the other 2 groups.

**Figure 1:**
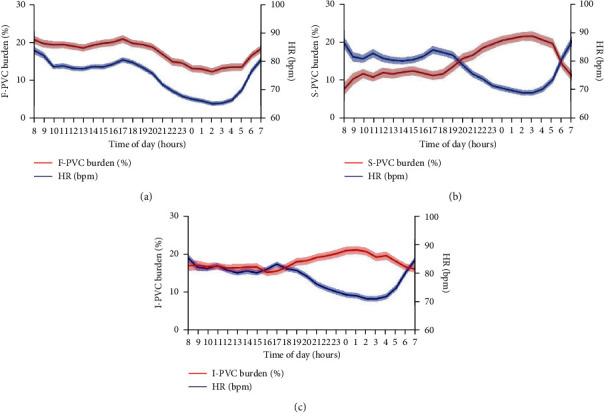
Circadian variability of PVC burden. Circadian rhythms of PVC burdens (as a percentage) and HR in F-PVC (a), S-PVC (b), and I-PVC (c). HR, heart rate; PVC, premature ventricular complexes.

The average hourly PVC burdens with the corresponding average hourly HR are shown in Figure 1. F-PVC (Figure 1(a)) typically showed a dipping pattern with a diurnal plateau and two surges at 8 am and 5 pm and an early morning nadir at 2 am. In contrast, S-PVC (Figure 1(b)) displayed a mirror-like pattern against the circadian fluctuation of HR, with a diurnal lowland and a steep climb from 6 pm to 3 am followed by a sudden drop to the morning nadir. I-PVC (Figure 1(c)), on the other hand, showed a constant level of PVC burden throughout the day. Despite heavier burden at night, the peak hour of I-PVC did not correspond with HR nadir, suggesting a different pattern from S-PVC. Of note, while both S-PVC and I-PVC exhibited nocturnal predominance, the day-and-night differences in PVC burden were smaller in I-PVC, suggesting its decreased circadian fluctuation and HR independency.

**Figure 2:**
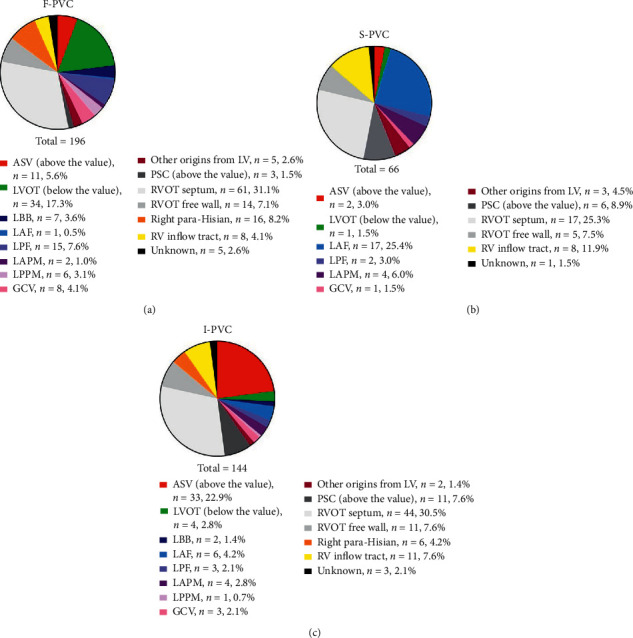
Origin distributions for PVC circadian patterns. Origin distributions for F-PVC (a), S-PVC (b), and I-PVC (c), respectively. ASV, aortic sinus of Valsalva; GCV, great cardiac vein; LAF, left anterior fascicle; LAPM, left anterior papillary muscle; LBB, left bundle branch; LPF, left posterior fascicle; LPPM, left posterior papillary muscle; LV, left ventricle; LVOT, left ventricular outflow tract; PSC, pulmonary sinus cusp; PVC, premature ventricular complexes; RV, right ventricle; RVOT, right ventricular outflow tract.

As shown in Figure 2, during mapping and ablation, RVOT was determined as the most common PVC source regardless of the circadian pattern, while origins from left ventricles exhibited prominently different distributions among groups. PVCs originating from left fascicular systems revealed heterogeneous distributions among groups. Taking LAF PVC as an example, only 1 case was categorized as F-PVC, while up to 16 cases were categorized as S-PVC. In addition, LVOT PVC constituted 17.3% of F-PVC, but only 1.5% and 2.8% of S-PVC and I-PVC, respectively. However, the adjacent ASV origin accounted for only 5.6% of F-PVC and 3.0% of S-PVC, but nearly one-fourth of I-PVC.

**Figure 3:**
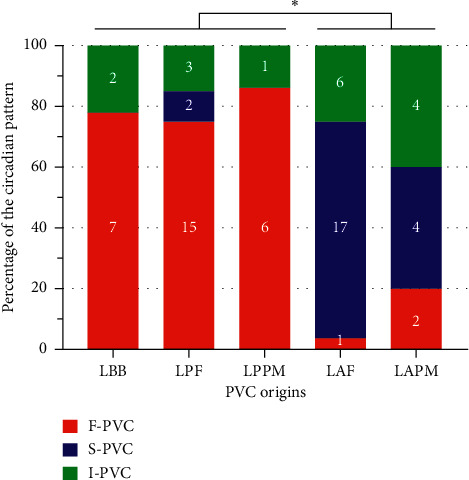
Differences in circadian patterns among PVCs of left fascicular origin. Circadian pattern distributions for PVCs arising from left fascicular systems and left papillary muscles. ^*∗*^Indicates *p* < 0.05. LAF, left anterior fascicle; LAPM, left anterior papillary muscle; LBB, left bundle branch; LPF, left posterior fascicle; LPPM, left posterior papillary muscle.

The heatmaps of hourly PVC frequencies from different anatomic origins were plotted (Supplemental Figure 3). As shown in Supplemental Figures 3(a) and 3(b), PVCs originating within the left Purkinje networks, including left papillary muscles, exhibited fascicular-specific distributions of circadian patterns.  Figure 3 summarizes that F-PVC was predominant in LBB, LPF, and LPPM origins, exhibiting evidently heightened arrhythmogenicity during waking than sleeping hours. In contrast, S-PVC predominated among LAF PVCs and was also common in LAPM origin, suggesting increased vulnerability to arrhythmias at night. Because human data suggested hemibranch-specific PVC circadian patterns, a set of rabbit studies was performed to evaluate if LAF and LPF Purkinje cells differed in electrophysiological properties, which might contribute to the arrhythmogenic heterogeneity in fascicular PVCs. As shown in Supplemental Figure 4, the TMP recording of Purkinje cells exhibited spontaneous phase 4 depolarization (red arrows) from both LAF and LPF. Compared with those in LPF, action potential duration (APD_80_) was shorter and the slope of spontaneous diastolic depolarization was steeper in LAF. Because the threshold of phase 0 activation was similar between the two hemibranches, spontaneous rhythm was significantly faster (shorter RR intervals) in LAF than LPF. These results may provide a clue to explain the heterogeneous circadian behaviors in PVCs with different origins.

**Figure 4:**
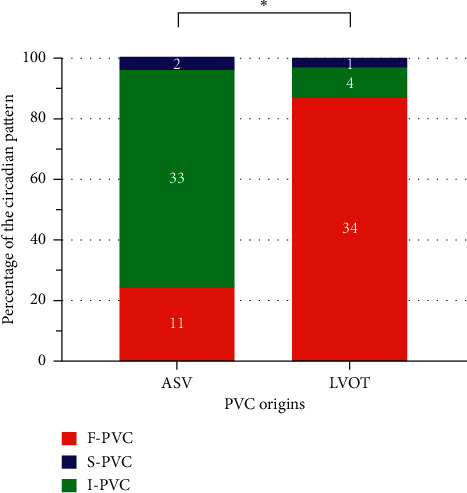
Differences in circadian patterns between ASV and LVOT PVCs. Circadian pattern distributions for ASV and LVOT PVCs. ^*∗*^Indicates *p* < 0.05. ASV, aortic sinus of Valsalva; LVOT, left ventricular outflow tract; PVC, premature ventricular complexes.

**Figure 5:**
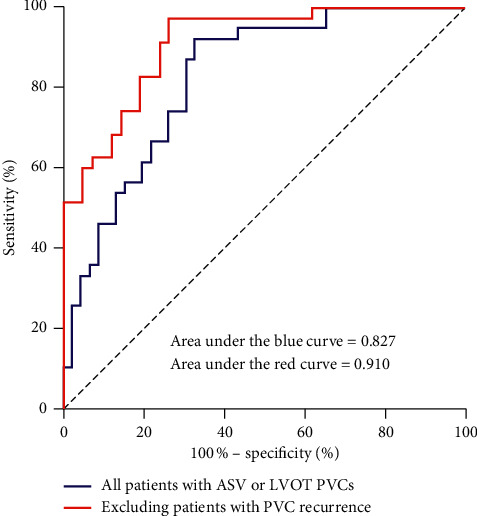
ROC curve documenting the ability of diurnal/nocturnal PVC burden ratio to differentiate LVOT and ASV origins. ROC curves plotting the true-positive rate (sensitivity) versus false-positive rate (1 − specificity) documenting the ability of diurnal/nocturnal burden ratio to differentiate LVOT and ASV origins in all patients (blue curve, AUC = 0.827) and in patients without PVC recurrence after ablation (red curve, AUC = 0.910). The ROC curve in combination with Youden's index supports a diurnal/nocturnal burden ratio of 0.92 as a cutoff. ASV, aortic sinus of Valsalva; AUC, area under the curve; LVOT, left ventricular outflow tract; PVC, premature ventricular complexes; ROC, receiver-operating characteristic.

**Figure 6:**
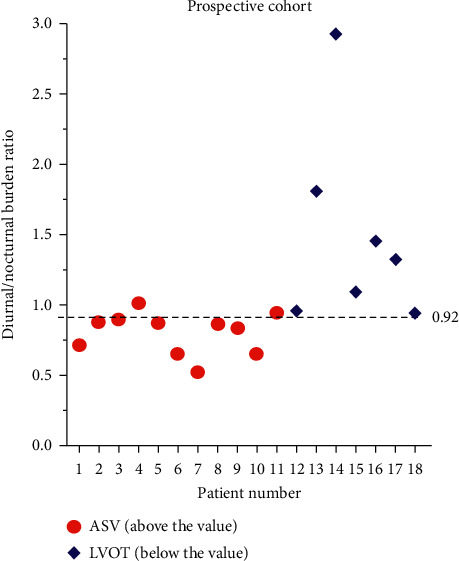
Scatter plot of the diurnal/nocturnal PVC burden ratio in the prospective cohort consisting of ASV and LVOT PVCs. Using a 0.92 cutoff successfully predicted PVC origin in 16/18 cases in a prospective cohort. ASV, aortic sinus of Valsalva; LVOT, left ventricular outflow tract; PVC, premature ventricular complexes.

Prominently different circadian pattern distributions from ASV and LVOT origins, the 2 adjacent anatomic sites, were also noticed (Supplemental Figures 3(c) and 3(d)). As shown in Figure 4, I-PVC was the major pattern for ASV origin (33/46, 72%), exhibiting higher nocturnal than diurnal frequencies. In contrast, F-PVC was predominant in LVOT origin (34/39, 87%). Despite the prominent differences, simply using the PVC circadian pattern failed to accurately distinguish LVOT from ASV origin because F-PVC still accounted for 24% of ASV PVCs. However, a diurnal/nocturnal PVC burden ratio ≥0.92 predicted an LVOT PVC with 92% sensitivity and 67% specificity (Figure 5, blue curve). After further excluding 8 cases with PVC recurrence (4 cases of ASV PVCs and 4 cases of LVOT PVCs) during the 3-month follow-up, the sensitivity and specificity increased to 97% and 74%, respectively, with a positive predictive value of 76% and a negative predictive value of 97% (Figure 5, red curve). The utility of the diurnal/nocturnal PVC burden ratio was further examined in a prospective cohort of 18 cases of PVCs originating from either ASV (*n* = 11) or LVOT (*n* = 7) who underwent successful ablation (Supplemental Figure 5; clinical characteristics in Table 2). Using diurnal/nocturnal PVC burden ratio to predict LVOT versus ASV origin achieved 89% (16/18) accuracy (Figure 6).

As further shown in the heatmaps (Supplemental Figure 3), PVC arising from other regions of LV or epicardial GCV did not show constant circadian patterns among patients. Unlike the cases with left-sided origins, PVC circadian pattern distributions were similar among RV origins. Of note, PVCs arising from RVOT, which is the most common origin of idiopathic PVCs, did not show distinct circadian patterns.

## 4. Discussion

In this study, we present a spectrum of circadian variability patterns for idiopathic monomorphic PVCs with different sites of origin. We found that circadian patterns of PVCs are (1) constant in most cases; (2) heterogeneous within left fascicular origins; and (3) different between ASV and LVOT sources. While 12-lead ECG criteria are widely used in identification of PVC origins, the circadian pattern might inform localization of PVC arising from the left ventricle. A 24-hour Holter monitoring provides additional clues of the anatomic sites of the origin and functional behavior of the arrhythmias.

### 4.1. Circadian Behavior of PVCs

The biorhythmic periodicity of the heart is governed concomitantly by the central SCN clock and the local clock within the heart [14]. The dyssynchronous rhythmicity between the two clocks may serve as a driver of ventricular arrhythmias [14]. Heterogeneity of action potential repolarization during circadian fluctuation displayed as heightened QT/QT_c_ dispersion and a shortened ventricular refractory period after awakening may precipitate the morning surge in ventricular arrhythmogenesis [4]. On the other hand, the cellular clock is also entrained by the SCN clock via neurohumoral modulation, such as sympathetic and parasympathetic nervous activities [14]. In addition, environmental misalignment also contributes to cardiac physiological dysfunction [15]. The concept of typical morning arrhythmic surge has recently been challenged by the observations of unexpected shift in arrhythmogenic circadian variation, suggesting that external factors such as the use of antiarrhythmic drugs and social jetlag of modern lifestyles also matter [3, 16].

In this study, the PVC circadian pattern was constant over relatively long periods in most cases, suggesting the existence of an endogenous mechanism for PVC circadian vulnerabilities. The interaction between the master and the cardiac clocks might differ among the 3 PVC circadian patterns. Spontaneous or drug-induced sympathetic and vagal activities are found prior to the onset of F-PVC and S-PVC, respectively, suggesting the predominance of central over local clock in those two types [9, 10]. However, the dyssynchronization of I-PVC from HR reflects its independence from autonomic innervation, which suggests a vital role of the intrinsic cardiac cellular clock. There also is molecular evidence that the dependence of cardiac gene expression on *β*-adrenergic activity differs among types of potassium channels [6]. Therefore, fluctuating combinations of participation between central and cardiac clocks dynamically modulate ion current properties and subsequently shape action potential morphology, duration, heterogeneity, and refractoriness, thus predisposing to the circadian oscillating vulnerabilities to ventricular arrhythmias. The class and the timing of antiarrhythmic medications (also termed chronotherapy) should be strategized in treating patients with initial or recurrent PVCs based on different circadian patterns.

### 4.2. ASV and LVOT PVCs

Despite anatomical vicinity, LVOT PVCs exhibit a distinct circadian pattern (F-PVC predominance) from ASV PVCs (I-PVC predominance). Miller and colleagues previously used coupling interval variability (ΔCI) to differentiate ASV/GCV sources from other outflow tract PVCs and further postulated a source-sink mismatch mechanism, which might also be the reasonable explanation of our current finding [17]. Briefly, due to limited amount of musculature extensions at the base of aortic root, the ectopic foci within the ASV (source) could easily overcome the activation threshold of the repolarized surrounding cells (sink) and are thus more likely to follow their own pattern (larger ΔCI and I-PVC). On the other hand, when surrounded by increased numbers of cells, the ectopic foci below the valve would find it more difficult to conquer the source-sink mismatch, therefore exhibiting a more externally controlled rhythm (smaller ΔCI and F-PVC) [17]. However, we did not find I-PVC predominance in GCV and PSC sources, which suggest that other mechanisms might also contribute, such as regional differences in cell types, ion channel expression, conduction, and autonomic innervation. In this study, we further introduced a diurnal/nocturnal PVC burden ratio as a highly sensitive diagnostic tool in distinguishing LVOT from ASV origin. This ratio could be calculated from a Holter report, thus facilitating its utilization in clinical practice.

### 4.3. PVCs from Left Fascicular Systems

The mechanism of fascicular-originated ventricular arrhythmias is highly debated. While several studies reported focal triggers in LAF arrhythmias, Nogami et al. proposed, as was further supported by other investigators, that macroreentry is dominant in fascicular arrhythmogenesis [18]. However, most of those observations are based on LPF VT (accounting for 90% of the cases), and LAF VT is much less common (<10%) [18, 19]. Of note, LAF PVCs are not rare but less frequently give rise to VT. In contrast, LPF VT is commonly observed in patients without frequent PVCs [20]. Taken together, these results suggest that different mechanisms might underlie LAF and LPF arrhythmogenesis.

In this study, we documented fascicular-specific PVC circadian patterns with LAF PVCs being slow-dependent and LPF PVCs showing F-PVC predominance. Subsequent TMP recordings of Purkinje fibers in rabbit hearts revealed heightened automaticity and shortened APD in LAF than LPF. Although there is lack of direct evidences, we postulate highly prevalent patterns of automaticity in LAF and reentry in LPF PVCs. The automaticity of both LAF and LPF is overdrive suppressed by the sinus node during the daytime. During fast sinus rhythm, some Purkinje cells are depolarized, while others are still within the refractory period, leading to functional block. With longer APD (and thus refractoriness) and more diffuse architecture than LAF [20], LPF harbors a more vulnerable substrate to reentrant circuits, leading to frequent PVCs during the daytime. At night, the slow sinus rhythm allows sufficient repolarization of LPF fibers to eliminate the functional block and subsequent reentry. In contrast, reentry might be less common in LAF PVCs because of thinner anatomic structure and shorter APD. However, due to the attenuation of the overdrive suppression by the sinus rhythm at night, the spontaneous diastolic depolarization of LAF competes with the excitation propagated from the sinus node in driving the ventricles, resulting in frequent nocturnal PVCs.

## 5. Limitations

First, patients' activities were not recorded, and thus, whether some suffered from chronic shift work, sleep disorder, or nocturnal light exposure was unknown. In this study, we found several F-PVC patients exhibiting reverse dipping patterns of HR with corresponding heightened nocturnal PVC burden. The utility of diurnal/nocturnal PVC burden ratio might be compromised in those patients, requiring combination with other 12-lead ECG algorithms to improve specificity in distinguishing LVOT from ASV sources. The utility of this ratio might also be limited, since there is only a small effort to map above or below the aortic valves during the ablation procedures. The clinical value of discerning RVOT from LVOT PVCs would be higher, but the diurnal/nocturnal burden ratio is not able to differentiate those PVC sources, given the data presented in this study. Second, other factors may also contribute to the differing circadian patterns between LAF and LPF origins, including Purkinje muscle coupling, ion current composition, autonomic innervation, and Ca^2+^-related triggered activities, which were not investigated in this study. Moreover, the circadian patterns vary in PVCs of the same origin, such as RVOT, suggesting the influence of other clinical and molecular factors. Furthermore, studies are needed to investigate those issues.

## 6. Conclusions

Circadian variability of PVCs arising from left ventricles exhibited origin specificity. PVC circadian patterns differ between the 2 left fascicular hemibranches and between LVOT and ASV origins. Using diurnal/nocturnal PVC burden ratio may help to differentiate PVCs from LVOT and ASV.

## Figures and Tables

**Table 1:** Characteristics of patients in the retrospective cohort.

	F-PVC (*n* = 196)	S-PVC (*n* = 68)	I-PVC (*n* = 144)
Age (years)	54 ± 18	47 ± 18^*∗*^	52 ± 18
Male	107 (55)	42 (62)	92 (63)
Symptomatic	176 (90)	60 (88)	128 (89)
Syncope	6 (3)	5 (7)	4 (3)
Nonsustained VT	47 (24)	13 (19)	28 (19)
Hypertension	79 (40)	20 (29)	57 (40)
Diabetes	18 (9)	6 (9)	14 (10)
Coronary artery disease	14 (7)	2 (3)	17 (10)
Atrial fibrillation	7 (4)	4 (6)	6 (4)
Malignant tumor	7 (4)	2 (3)	4 (3)
Left ventricular ejection fraction (%)	61 ± 5	60 ± 6	60 ± 7

Data are presented as mean ± SD or *n* (%) ^*∗*^vs. F-PVC, *p* < 0.05. PVC, premature ventricular complexes; VT, ventricular tachycardia.

**Table 2:** Characteristics of patients with ASV or LVOT PVCs in the prospective cohort.

Patient characteristics	*n* = 18
Age (years)	58 ± 15
Male	9 (50)
Symptomatic	17 (94)
Hypertension	8 (44)
Diabetes	1 (6)
Coronary artery disease	1 (6)
Atrial fibrillation	2 (11)
Left ventricular ejection fraction (%)	63 ± 9

Data are presented as mean ± SD or *n* (%). PVC, premature ventricular contraction; ASV, aortic sinus of Valsalva; LVOT, left ventricular outflow tract.

## Data Availability

The data used to support the findings of this study are available from the corresponding author upon request.

## Supplementary Materials

Supplementary Materials Detailed methods and figures are further described in the Supplementary materials.
