# Supplementary material for: Utility of Circadian Variability Patterns in Differentiating Origins of Premature Ventricular Complexes
Source: J Interv Cardiol. 2020 Oct 30;2020:7417912. doi: 10.1155/2020/7417912 (PMC7647775; doi:10.1155/2020/7417912)
Supplement: Supplementary Materials — Detailed methods and figures are further described in the Supplementary materials. [file 7417912.f1.docx]

**Utility of Circadian Variability Patterns in Differentiating Origins of Premature Ventricular Complexes**

Mu Chen, *MD*,^a^ Qunshan Wang, *MD*,^a^ Jian Sun, *MD*,^a^ Peng-Pai Zhang, *MD*,^a^ Wei Li, *MD*,^a^ Rui Zhang, *MD*,^a^ Bin-Feng Mo, *MD*,^a^ Tai-Zhong Chen, *MD*,^a^ Juyi Wan, *MD*,^b^ Dong-Zhu Xu, *MD*,^c^ Kazutaka Aonuma, *MD, PhD*,^c^ Yi-Gang Li, *MD,*^a^

^a^ Department of Cardiology, Xinhua Hospital, School of Medicine, Shanghai Jiao Tong University, Shanghai, China. ^b^ Department of Cardiothoracic Surgery, Affiliated Hospital of Southwest Medical University, Luzhou, China. ^c^ Cardiovascular Division, Institute of Clinical Medicine, Faculty of Medicine, University of Tsukuba, Japan.

**Address for Correspondence:**

Dr. Yi-Gang Li, [liyigang@xinhuamed.com.cn](mailto:liyigang@xinhuamed.com.cn)

**SUPPLEMENTAL MATERIAL**

**Supplemental methods**

**Patient selection**

All patients in this study were enrolled from Xinhua Hospital, School of Medicine, Shanghai Jiao Tong University, Shanghai, China. The Institutional Review Board committee approved the retrospective and prospective studies.

We retrospectively reviewed records of all patients who underwent radiofrequency catheter ablation of idiopathic premature ventricular complexes (PVCs) between May 2008 and July 2018. Only patients with at least one 24-hour Holter-monitoring documented PVCs >3000 beats/24h prior to the procedure were studied. Cases with pleomorphic PVCs, sustained ventricular tachycardia (VT) or inconsistent circadian patterns among repeated Holter recordings were excluded. While most patients had normal left ventricular function by echocardiography, patients with cardiomyopathy were excluded if the PVCs were suspected to be secondary to the underlying cardiomyopathy, such as myocardial infarction and severe valvular disease. Cases with presumed cardiomyopathies due to high PVC burdens were included.

Because the retrospective evaluation revealed different circadian patterns between PVCs arising from aortic sinus of Valsalva (ASV, above the aortic valve) and left ventricular outflow tract (LVOT, below the aortic valve), a second cohort of patients whose PVC origin was predicted as left-sided with outflow tract morphology by 12-lead ECG ^1^ were prospectively enrolled to assess the utility of our proposed Holter algorithm in differentiation of those two adjacent sources. We excluded 1 patient whose PVC origin was predicted as LVOT but was successfully ablated at right ventricular outflow tract (RVOT).

In addition, patients’ medical history, blood tests, transthoracic echocardiography and 12-lead surface ECG both during sinus rhythm and in the presence of PVCs were collected and reviewed.

**PVC circadian patterns**

At least one 24-hour Holter recording was acquired before the procedure with at least 48h discontinuation of antiarrhythmic drugs. Patients were instructed to maintain normal daily activities and keep sleeping arrangements during the recordings.

Three types of circadian variability patterns were categorized based on the Pearson’s correlation between hourly PVC counts and hourly average mean HR, as prescribed previously ^2^. In brief, if the correlation was positive with statistical significance (Pearson, p<0.05), such circadian pattern was defined as F-PVC. If the correlation was significantly negative (p<0.05), such pattern was named as S-PVC. If no statistical significance was achieved (p>0.05), PVC pattern was considered as I-PVC.

For each patient, the hourly PVC counts were ranked and plotted in a line of heatmap (24 color gradients) with an x-axis of 24-hour timeline. The hour with highest PVC frequency was plotted as red and the hour with lowest PVC frequency as blue. The hours with PVC frequencies in between were presented by colors gradually shifting from red to blue with 24 gradients according to their ranks in 24 hours. In addition, PVC burden was calculated by computing PVC counts divided by total heart beats within a certain period. Specifically, diurnal (daytime) PVC burden was calculated from 8 am to 8 pm (12 hours), while nocturnal (nighttime) burden was acquired between 8 pm to 8 am of the next day (12 hours). A diurnal/nocturnal PVC burden ratio was subsequently calculated in PVCs with ASV and LVOT origins.

**Mapping and ablation**

Antiarrhythmic medications were routinely discontinued at least 48h before the procedure. Informed consent was obtained from all patients. None or minimal sedation strategy was used to avoid potential PVC suppression. A standard diagnostic electrophysiological study was performed using several multielectrode catheters. In patients with sufficient intraprocedural ectopy, mapping of the PVC origin was performed by targeting the earliest local bipolar activation time compared with the surface QRS of the PVC. In cases with infrequent intraprocedural PVCs, pace mapping at a threshold just above local capture was attempted and a matching pace map was identified when ≥ 10/12 leads were matched between the paced QRS complex and the clinical PVC. After excluding an unacceptable proximity to a major coronary artery, ablation was attempted using standard or irrigated radiofrequency energy under the guidance of CARTO (Biosense Webster, Diamond Bar, CA, USA) or ENSITE (St. Jude Medical, St Paul, MN, USA) electroanatomic mapping systems in addition to standard fluoroscopy. A nonirrigated ablation catheter with power delivered up to 50 W and temperature up to 50 ºC or an irrigated catheter with maximal power setting of 30 to 35 W were used for most of the cases. For PVCs originating within the cardiac venous system or in vicinity of His bundle, power delivery was titrated to avoid high impedance and impairment of the normal conduction system. The site of successful ablation was identified as the one that led to the abolition of the clinical PVCs at 30 min after the last radiofrequency delivery, both with and without isoproterenol stimulation.

Fifteen sites of PVC origin were classified as follows: ASV, LVOT, left bundle branch (LBB), left anterior fascicle (LAF), left posterior fascicle (LPF), left anterior papillary muscle (LAPM), left posterior papillary muscle (LPPM), great cardiac vein (GCV), other origins from left ventricles, pulmonary sinus cusp (PSC), the septum of RVOT, free wall of RVOT, para-Hisian region, right ventricular inflow tract and tricuspid annulus (except the para-Hisian region). Sources of PVCs with immediate failure of ablation were designated as of “unknown” origin.

**Animal studies**

Because our human data suggested hemibranch-specific PVC circadian patterns, we further performed a set of rabbit studies to evaluate if electrophysiological properties differed between LAF and LPF Purkinje cells, which might contribute to the heterogeneous circadian behaviors of fascicular PVCs. The animal study was approved by the Institutional Animal Care and Use Committee (IACUC) of Xinhua Hospital, and conformed with the Guide for the Care and Use of Laboratory Animals. A total of 15 adult (5-6 months old, 8 males, 3.06-3.32 kg) New Zealand white rabbits were used (Slaccas Experimental Animal Company, Shanghai, China). The transmembrane potentials (TMPs) of Purkinje fibers were recorded using a technique similar to that reported previously ^3^. In brief, after euthanasia by overdose intravenous sodium pentobarbitone (160 mg/kg), the hearts were removed and Langendorff perfused with Tyrode’s solution (in mmol/L: NaCl 125, NaHCO_3_ 24, NaH_2_PO_4_ 1.8, MgCl_2_ 0.5, CaCl_2_ 1.8. KCl 4 and glucose 5.5, bubbled with 95% O_2_ and 5% CO_2_, pH 7.40 at 37 ºC). The left ventricle anterior wall was subsequently cut open to expose the septum. LAF and LPF were recognized below the anatomical bifurcations of the left bundle branch with Purkinje networks loosely attached to the septal endocardium. Under the microscope, Purkinje fibers were carefully and gently separated from their attached endocardium with their two ends connecting to a small amount of endocardial tissue. The Purkinje fibers were then fixed at both ends and placed in a chamber superfused with Tyrode’s solution. TMPs were recorded at the distal portion of the Purkinje fibers using standard capillary glass microelectrodes filled with 3 mol/L KCl with tip resistance of 20 MΩ. Axoscope software was used for data acquisition and action potential parameter measurements. Stable recordings were compared between LAF and LPF from the same rabbit.

**Statistical analysis**

Continuous and ordinal variables are shown as mean ± SD and nominal variables as n (%) as appropriate, unless mentioned specifically. Between-group differences in nominal variables were assessed using Pearson’s chi-square tests or Fisher exact tests. Differences in continuous variables among groups were assessed using a one-way ANOVA with Tukey post-tests. Paired Student’s t tests were used to compare 2 variables obtained from the same patient or rabbit. A receiver operating characteristic curve was generated for sensitivity and specificity analyses, and Youden’s index applied to determine the optimal cutoff for diurnal/nocturnal PVC burden ratio as a diagnostic test. Statistical analyses were performed using SPSS 23.0 (IBM, Armonk, NY, USA). A 2-sided P value <0.05 was considered statistically significant.

**Supplemental references**

1. Betensky BP, Park RE, Marchlinski FE, Hutchinson MD, Garcia FC, Dixit S, et al. The V(2) transition ratio: a new electrocardiographic criterion for distinguishing left from right ventricular outflow tract tachycardia origin. Journal of the American College of Cardiology 2011; 57: 2255-2262, doi:10.1016/j.jacc.2011.01.035.

2. Hamon D, Abehsira G, Gu K, Liu A, Blaye-Felice Sadron M, Billet S, et al. Circadian variability patterns predict and guide premature ventricular contraction ablation procedural inducibility and outcomes. Heart rhythm 2018; 15: 99-106, doi:10.1016/j.hrthm.2017.07.034.

3. Wan J, Chen M, Wang Z, Everett THt, Rubart-von der Lohe M, Shen C, et al. Small-conductance calcium-activated potassium current modulates the ventricular escape rhythm in normal rabbit hearts. Heart rhythm 2019; 16: 615-623, doi:10.1016/j.hrthm.2018.10.033.

**Online Figures**

**Online Figure 1**. **PVC circadian patterns among repeated Holter recordings before ablation**

**
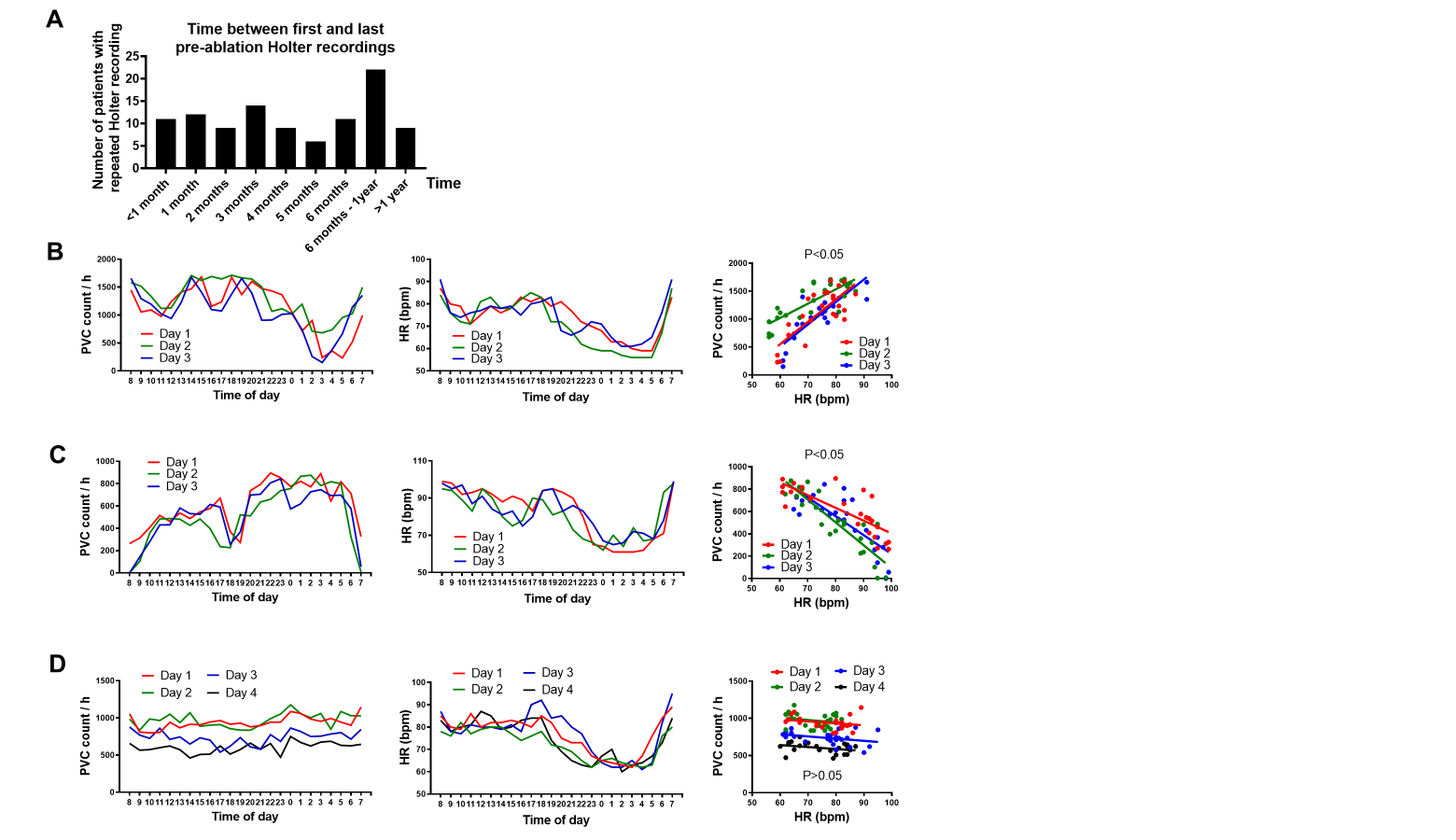
**

**A.** Distributions of the time differences between the first and the last pre-ablation Holter recordings. Representative cases of F-PVC (**B**), S-PVC (**C**) and I-PVC (**D**) with repeated pre-ablation Holter recordings showing hourly PVC counts (left panel), hourly mean HR (middle panel) and their correlations (right panel), revealing the high consistency of PVC circadian patterns among separated recording days. HR = heart rate; PVC = premature ventricular complexes.

**Online Figure 2**. **Enrollment of patients into the retrospective cohort.**

**
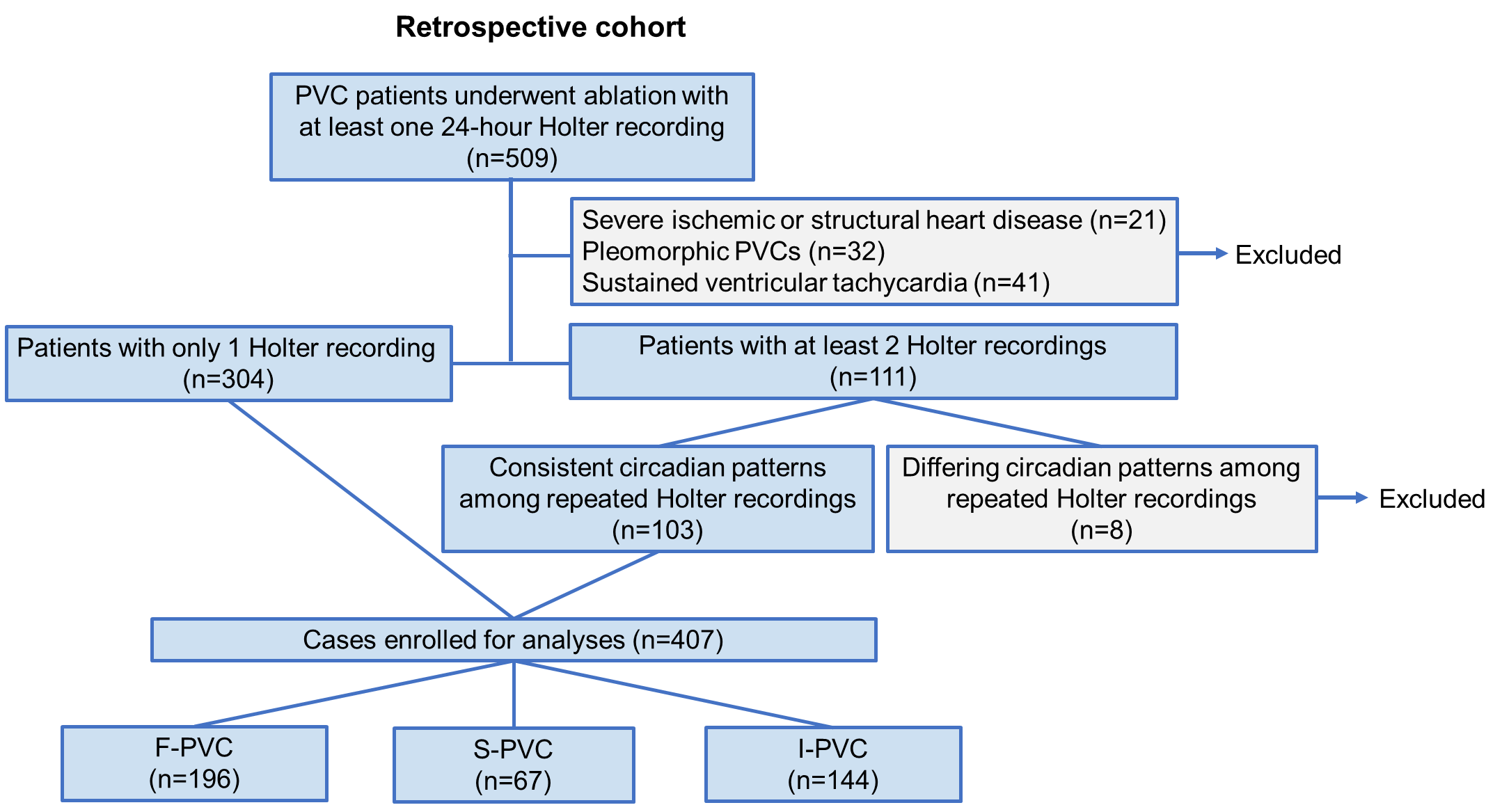
**

The retrospective cohort enrolled patients with monomorphic idiopathic PVCs who underwent catheter ablation with at least 1 pre-ablation Holter recording. PVC = premature ventricular complexes

**Online Figure 3. Heatmaps of PVC frequencies from different origins.**

**
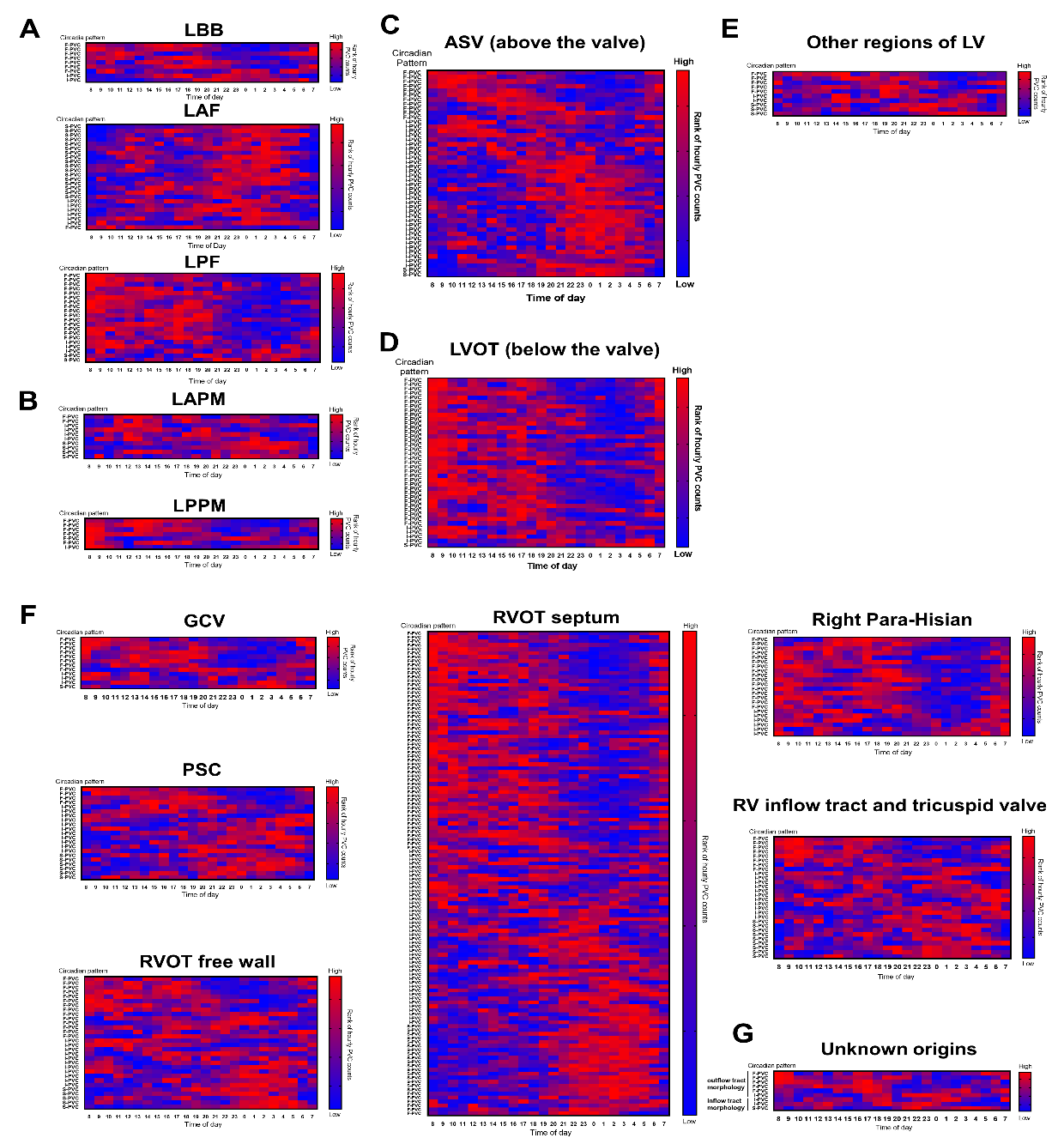
**

Heatmaps displayed the rank of hourly PVC frequencies from different origins (**A-G**). Each row represents an individual. Red and blue indicate the high and low ranks of the hourly PVC counts, respectively. ASV = aortic sinus of Valsalva; GCV = great cardiac vein; LAF = left anterior fascicle; LAPM = left anterior papillary muscle; LBB = left bundle branch; LPF = left posterior fascicle; LPPM = left posterior papillary muscle; LV = left ventricle; LVOT = left ventricular outflow tract; PSC = pulmonary sinus cusp; PVC = premature ventricular complexes; RV = right ventricle; RVOT = right ventricular outflow tract.

**Online Figure 4. Transmembrane potentials of rabbit Purkinje cells.**

**
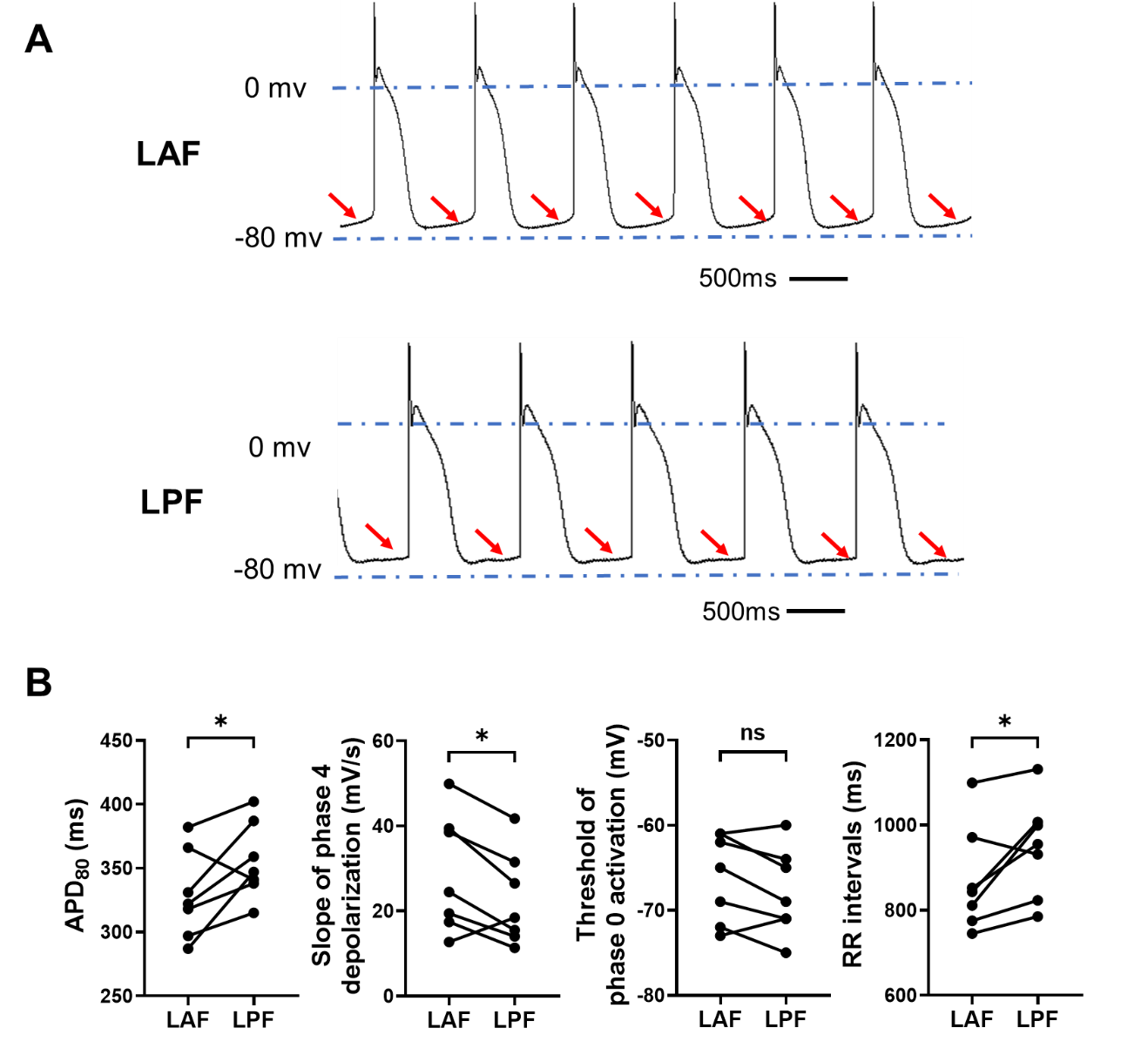
**

Animal study to reveal differences in electrophysiological characteristics between two left fascicular hemibranches. **A.** Representative transmembrane potential (TMP) recordings in Purkinje cells from LAF and LPF of the same rabbit. Red arrows indicate spontaneous phase 4 depolarization. **B** shows summary data on electrophysiological parameters of Purkinje cells from LAF and LPF, including action potential duration (APD_80_), slope of phase 4 depolarization, threshold of phase 0 activation and RR intervals. Student’s paired t-tests, * indicates p<0.05. LAF = left anterior fascicle; LAPM = left anterior papillary muscle; LBB = left bundle branch; LPF = left posterior fascicle; LPPM = left posterior papillary muscle

**Online Figure 5. Prospective cohort of patients with ASV or LVOT PVCs.**

**
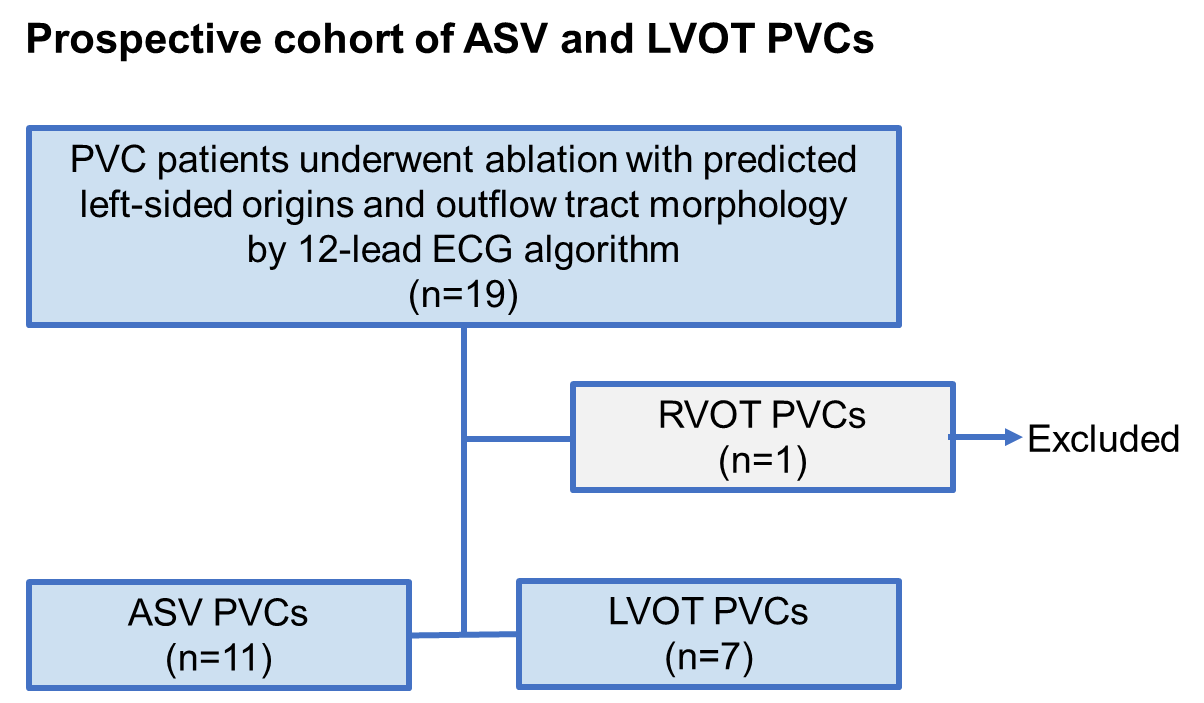
**

The prospective cohort enrolled patients with ASV or LVOT PVCs aiming to assess the predictive value of diurnal/nocturnal PVC burden ratio in differentiation of the two adjacent sources. ASV = aortic sinus of Valsalva; ECG = electrocardiogram; LVOT = left ventricular outflow tract; PVC = premature ventricular complexes; RVOT=right ventricular outflow tract.
